# Visit characteristics of emergency departments caring for persons living with dementia: a nationally representative sample

**DOI:** 10.1002/alz70860_096855

**Published:** 2025-12-23

**Authors:** James Galske, Jonathan Martindale, Julie Bynum, Arjun Venkatesh, Karina Berg, Cameron Gettel

**Affiliations:** ^1^ University of Connecticut School of Medicine, Farmington, CT, USA; ^2^ University of Michigan, Ann Arbor, MI, USA; ^3^ Yale School of Medicine, New Haven, CT, USA; ^4^ University of Connecticut Health Center, Farmington, CT, USA

## Abstract

**Background:**

Emergency Department (ED) visits among persons with Alzheimer's Disease and Related Dementias (ADRD) have increased in recent years. Despite growing research focused on this population in the ED setting, limited knowledge exists about the key risk factors associated with greater ED utilization. Understanding demographic factors linked to ED visits and identifying EDs frequently managing ADRD patients is essential for developing interventions and optimizing resource allocation. This study aims to quantify the proportion of ED visits by persons with ADRD and identify patient‐ and ED‐level characteristics associated with increased ED utilization.

**Method:**

We conducted a cross‐sectional study using 2019 Medicare Part B claims data to quantify ED visits by Medicare beneficiaries with ADRD. We identified persons with ADRD using the validated 1‐year BynumStandard Algorithm. We analyzed demographic characteristics, including age, sex, race and ethnicity, dual‐eligibility status, and ED‐level factors including geographic region, urbanization, hospital size, teaching status, private equity ownership, and geriatric accreditation. We assessed group differences using the Mann‐Whitney U and Kruskal‐Wallis tests.

**Result:**

Among 3,178 EDs, persons with ADRD accounted for a median of 15.6% of all ED visits by Medicare beneficiaries. Beneficiaries with ADRD accounted for a median of 32.2% of ED visits in those aged 85+, 17.8% in ages 75‐84, and 6.6% in ages 65‐74 (*p* <0.0001). Separately, among Medicare‐Medicaid dual eligible beneficiaries, those with ADRD accounted for a median of 30.6% of ED visits, compared to 12.8% among the Medicare alone population (*p* <0.0001). The median proportion of ED visits by beneficiaries with ADRD varied by hospital size (large 15.6% vs. medium 15.9% vs. small 14.8%; *p* <0.0001), teaching status (yes 14.8% vs. no 15.7%; *p* = 0.0009), and geographic region (South 16.7% vs. Northeast 15.9% vs. Midwest 14.6% vs. West 13.5%; *p* <0.0001).

**Conclusion:**

Persons with ADRD were responsible for approximately 1 in 6 ED visits among Medicare beneficiaries, with disproportionately high reliance on ED care among older (85+) and dual‐eligible populations. Future research should explore the causes of these visits, particularly their relation to dementia, and assess how evolving dementia and primary care models influence ED utilization.

